# The combination of postnatal maternal separation and social stress in young adulthood does not lead to enhanced inflammatory pain sensitivity and depression-related behavior in rats

**DOI:** 10.1371/journal.pone.0202599

**Published:** 2018-08-24

**Authors:** Julien Genty, Milène Tetsi Nomigni, Fernand Anton, Ulrike Hanesch

**Affiliations:** Research group Stress, Pain and Pain Modulation, Institute for Health and Behavior, University of Luxembourg, Luxembourg, Luxembourg; University of California Los Angeles, UNITED STATES

## Abstract

The cumulative and match/mismatch hypotheses of stress are still under discussion regarding the effects of early life stress (ELS) on the vulnerability or resilience to psychopathology. In this context, an additional stress in later life (second hit) often leads to stress-related disorders that frequently include comorbid pain states. We previously observed that maternal separation (MS), a model of ELS, reduces vulnerability to neuropathic and inflammatory pain in rats. In the present study, we investigated the effects of an additional later stressor on the vulnerability to inflammatory pain. Sprague Dawley pups were divided into 4 groups: controls (CON, no stress), MS, social stress (SS) and MS+SS. At young adult age (from 7 to 15 weeks), stress- as well as pain-related parameters were evaluated prior and during 21 days following the induction of paw inflammation with complete Freund’s adjuvant (CFA). Finally spinal glutamatergic transmission, immunocompetent cells, pro-inflammatory cytokines and growth factors were examined using qPCR. None of the stress conditions had a significant impact on corticosterone levels and anhedonia. In the forced swim test, MS and SS displayed increased passive coping whereas the combination of both stressors revoked this effect. The different stress conditions had no influence on basal mechanical thresholds and heat sensitivity. At 4 days post-inflammation all stress groups displayed lower mechanical thresholds than CON but the respective values were comparable at 7, 10, and 14 days. Only on day 21, MS rats were more sensitive to mechanical stimulation compared to the other groups. Regarding noxious heat sensitivity, MS+SS animals were significantly less sensitive than CON at 10 and 21 days after CFA-injection. qPCR results were mitigated. Despite the finding that stress conditions differentially affected different players of glutamatergic transmission, astrocyte activity and NGF expression, our biochemical results could not readily be related to the behavioral observations, precluding a congruent conclusion. The present results do neither confirm the cumulative nor corroborate or disprove the match/mismatch hypothesis.

## Introduction

While chronic stress often leads to deleterious effects, it may also trigger adaptive changes that can possibly be beneficial. In this context, a major research emphasis was recently put on the long-term consequences of early life stress (ELS) for the processing of novel stress exposure in the course of adulthood. Several concepts aimed at predicting these programming effects have been advanced, namely the cumulative stress hypothesis, the inoculation and the match/mismatch hypothesis [1]. The accumulation of stressors throughout the lifespan may exceed a threshold and enhance the vulnerability to develop psychopathology. In the cumulative stress hypothesis early exposure to stressors is therefore regarded as detrimental. On the contrary, the inoculation hypothesis states that early exposure to stressors leads to a better coping. Finally, the match/mismatch theory states that beneficial consequences may be expected if individuals are exposed to similar (matching) adversities in adulthood while a mismatching environment may lead to enhanced vulnerability for psychopathologies [2].

Until recently, most studies investigated the brain mechanisms underlying the impact of ELS on vulnerability/resilience to stress-related cognitive and/or emotional impairment later in life [2–4]. Only few reports focused on altered vulnerability for potential comorbid pathologies such as chronic pain [5–7]. Although there are some data on the effects of ELS on neuropathy and inflammatory pain sensitivity [5,8] very little is known about the potential alterations of biochemical pathways involved in the regulation of spinal nociceptive processing. The spinal cord is however of major importance since it constitutes the point of entry and distribution of noxious information throughout the central nervous system [9]. We previously observed that maternal separation (MS), a rodent model of ELS could *per se* have a beneficial effect regarding the establishment of neuropathy- and inflammation-induced hyperalgesia in later life [10,11].

Considering the concepts on the enduring consequences of ELS for stress reactivity and affective disorders, we sought to determine whether ELS-treated rats displayed altered vulnerability for inflammatory pain and concomitantly altered processing of negative emotions when exposed to ongoing social stress (SS) in young adulthood. The second goal of our study was to investigate the ELS- and/or SS-dependent regulation of spinal biochemical markers involved in the processing of stress and nociception. For this purpose, we assessed stress-related alterations of the mRNA expression of spinal glucocorticoid receptors (GR) and of mediators involved in glutamatergic transmission. GR may play a critical role in the framework of chronic pain since they have been shown to be involved in the injury-induced upregulation of NMDA receptors [12,13]. NMDA receptors are crucial for central sensitization [14,15]. Furthermore, metabotropic group 1 glutamate receptors (mGluR), particularly mGluR1 and 5, have been shown to be implicated in increased excitability and central sensitization [16]. In addition, it was shown that ELS has enduring consequences on the functioning of immunocompetent cells. Indeed the priming of brain microglia by ELS may lead to an enhanced response to subsequent stress [17] and activate astrocytes. Consequently, we evaluated the spinal content of ionized calcium binding adaptor molecule 1 (Iba1) and glial fibrillary acidic protein (GFAP) as markers of microglial and astrocytic reactivity [18]. It is well established that activated glial cells have the ability to release several mediators, including pro-inflammatory cytokines that may in turn enhance the sensitivity of nociceptive neurons [19–22]. Among these mediators, spinal tumor necrosis factor alpha (TNF-α), interleukin-1β (IL-1β) and interleukin-6 (IL-6) are of major importance [23,24]. Finally, we investigated the mRNA expression of the neurotrophic factors nerve growth factor (NGF) and glial cell line-derived neurotrophic factor (GDNF) that have both been discussed to be implicated in chronic stress/depression as well as in nociception [25].

## Material and methods

### Animal housing

Sprague Dawley male rats were obtained from our in-house breeding facility. All animals were kept under standardized housing conditions in a temperature (22 ± 1°C), humidity (60 ± 10%) and light/dark cycle (7 am to 7 pm) controlled room. Food and water were provided *ad libitum*. Unless indicated differently, animals were handled twice per week during the cleaning of the cages.

The different manipulations carried out on the animals were performed in accordance with the Directive 2010/63/EU on the protection of animals used for scientific purposes and the ARRIVE guidelines were followed. The Animal Experimentation Ethics Committee (AEEC) of the University of Luxembourg and the "Ministère de l'Agriculture, de la Viticulture et de la Protection des Consommateurs" approved all experimental procedures (ID 15-SPM-01-UH).

### Breeding

Male and female Sprague Dawley rats used for the breeding were purchased from Harlan Laboratories (Netherland). After a week of habituation to the housing conditions, two females were mated with a male for 4 days. Gestating females were housed individually 15 days after the end of mating and checked twice daily for birth from day 18. The day of birth was defined as post-natal day 0 (P0). The offspring obtained from 16 litters ranging from 9 to 15 animals were used in the present experiments.

### Experimental procedure

**Fig 1:**
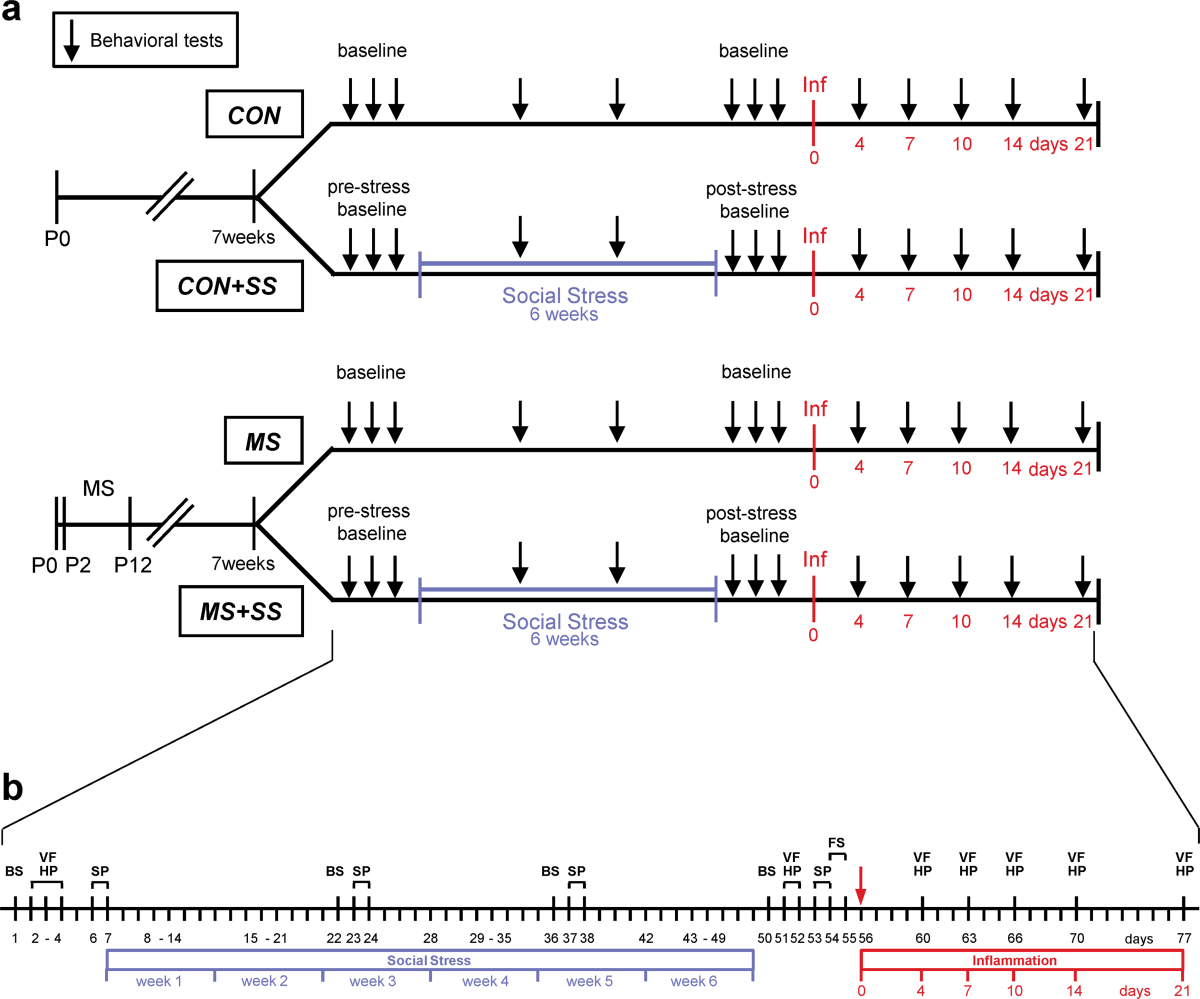
Experimental design. The course of the experiment is depicted in (a) and the experimental procedures carried out are outlined in detail in (b). After birth (P0), rats were separated into two experimental groups: the control group (CON) that was left undisturbed until the age of 7 weeks while the MS group that underwent the maternal separation from postnatal day 2 (P2) to 12 (P12). At 7 weeks, each of the two groups were subdivided into a non-social stress (CON, n = 23 resp. MS, n = 16) and a social stress condition (CON+SS, n = 12 resp. MS+SS, n = 12). Baseline values were collected for blood corticosterone levels (blood sampling, BS), basal mechanical (von Frey test, VF) and thermal (hot plate test, HP) sensitivity and for depressive behavior (sucrose preference test, SP). The animals in the CON+SS and the MS+SS groups were then subjected to the social stress paradigm for 6 weeks. Blood was taken and sucrose preference was measured during the social stress period after week 2 and 4. After the social stress, BS, VF, HP and SP were performed to reveal a post-stress baseline. Additionally, the animals were subjected to a forced swim test (FS). Following that, inflammation (Inf) was induced by Complete Freund’s Adjuvant in all four groups. Mechanical and thermal noxious sensitivities were further measured on days 4, 7, 10, 14 and 21. After the last testing, animals were sacrificed.

The experimental design of this study is graphically summarized in Fig 1.

Four groups of rats were used: CON (underwent neither MS nor SS, n = 23), MS (underwent MS but not SS, n = 16), SS (underwent SS but not MS, n = 12) and MS+SS (underwent the combination of MS and SS, n = 12). All animals were subjected to all the experimental procedures foreseen for the respective groups.

Apart from MS, rats were not subjected to any manipulation until the age of 6 weeks. At that stage they were habituated to the experimenter and experimental devices. At 7 weeks of age, which correspond to a late juvenile phase still prone to vulnerability for the development of psychopathologies [26], the experimental procedures started with blood sampling to measure basal corticosterone (CORT) levels and with behavioral tests to obtain baseline values. Mechanical (von Frey test) and thermal (hot plate test) noxious sensitivity as well as anhedonic (sucrose preference test) behavior were assessed. Once the pre-stress baseline was established, the animals in the relevant groups were subjected to the SS paradigm. Blood sampling and the sucrose preference test were also performed after 2 and 4 weeks of SS in order to evaluate the effect of stress across time. Following 6 weeks of SS, all animals underwent a second baseline testing (= post-stress baseline). Then, animals underwent the forced swim test (FST) in order to evaluate their coping strategy when facing uncontrollable stress. Subsequently, all animals were injected with Complete Freund's Adjuvant (CFA) to induce chronic inflammatory pain and noxious sensitivity was tested at post-injection days 4, 7, 10, 14 and 21. Finally, animals were euthanized and L4-L6 levels of the spinal cord were sampled to assess mRNA expression of spinal biomarkers involved in the modulation and the transmission of noxious signals.

### Maternal separation

ELS, as previously described in more detail in recent studies [6,10], was performed via the maternal separation (MS) procedure on at least four different litters. Every day, from *P2* to *P12*, pups were separated from the dams, placed on a heated pad having a temperature of 33 ± 2°C and left undisturbed for 3 hours. After each separation, the offspring were returned to their home cage with the dam. Rats from the experimental groups CON and SS that were not selected for the MS procedure were left undisturbed during this period.

### Social stress

The social stress (SS) paradigm used was based on studies performed in mice by Schmidt and colleagues [27,28] and on a model initially developed in rats by Mormede and colleagues [29]. Young siblings from 4 different litters were housed 3 per cage until they reached the age of 8 weeks. The group composition in the cages was then changed twice per week during cage cleaning, for a period of 6 weeks. The redistribution of the rats was performed in a manner ensuring a minimum of interactions between already familiarized rats and siblings. At the end of the SS period, rats were brought together with their original siblings. Animals that did not undergo the SS procedure were left undisturbed.

### Pain sensitivity assessment

Mechanical thresholds and thermal sensitivity testing were performed successively on the same day with a minimum of 30 min interval to minimize nociceptor sensitization.

#### Mechanical noxious sensitivity

Animals were left undisturbed in the testing room to acclimatize for at least one hour before being placed in a Plexiglas chamber with a wire mesh floor. Each hind-paw was alternatively tested 3 times with at least a 5 minutes interval. A mechanical stimulation device (dynamic plantar aesthesiometer; Ugo Basile) was applied on the plantar surface of the hind-paw to induce progressively increasing pressure via a blunted metallic filament (ramp of 50 g in 35 s). The pressure that evoked a withdrawal of the tested hind-paw was recorded automatically and was considered as the mechanical nociceptive threshold. The results obtained for each paw were averaged for each testing day. In order to establish the pre-stress baseline, animals were tested on 3 consecutive days, the thresholds obtained on each day were averaged and the mean was defined as the mechanical sensitivity threshold of the animal.

#### Thermal noxious sensitivity

The hot plate apparatus (Ugo Basile) composed of a flat metal plate enclosed by a Plexiglas cylinder was used to evaluate thermal noxious sensitivity. For the test, animals were placed on the hot plate (50 ± 1°C) and left undisturbed until they presented signs of pain. At the first clear signs of pain-related behavior, the test was stopped and the animals were removed from the hot plate. The latency (s) to display nocifensive behavior was considered to reflect heat sensitivity.

### Depressive-like behavior assessments

#### Sucrose preference test: Two bottle choice

Anhedonia is one of the traits observed in patients suffering from depression. In animal models of depression, the two bottle choice paradigm is commonly chosen to evaluate this behavior. This test was performed before, during (week 2 and 4) and after the SS period. During the test, each rat was housed individually for 24 hours and was given the possibility to drink from two bottles, one containing tap water and the other a 0.8% sucrose solution. In order to avoid a side preference effect, the bottles were randomly distributed between left and right sides. Food, water and sucrose solution were weighed before and after the 24 hours in order to obtain raw consumption levels. The preference was then calculated as the volume of sucrose solution ingested over the total volume consumed during the 24 hours of test.

%Preference=[(SucroseIntake/TotalIntake)x100]

For anhedonia to be confirmed, the sucrose consumption had to be below 65%. Values were removed if a bottle presented a damage provoking a leakage of sucrose solution or water.

#### Forced swim test (FST)

We used a modified version of the FST [30] which was performed only once at the end of the SS period since it puts a strain on the animals. It consisted in placing the rats in glass cylindrical tanks (diameter: 24cm, height: 45cm) containing water (23.5 ± 0.5°C, water depth 35 ± 2 cm) on 2 consecutive days. The water was changed after each swim test. As for other behavioral tests, animals were brought to the experimental room at least one hour before the testing protocol. On the first test day, animals were submitted to a 15 minutes swim (pre-test) session. The session on the second day consisted in a 5 min forced swim during which the time rats spent immobile, climbing or swimming was acquired. An animal was considered to be immobile only when floating or performing very slight movements allowing it to keep its head above water level. After the test sessions, rats were dried in a towel and returned to their home cage.

### Induction of peripheral inflammation and evaluation of CFA-induced edema

Rats were briefly anesthetized with isoflurane (4.3% for induction and 2.5% for maintenance) using an anesthesia unit (Univentor 400, Zejtun). Inflammation was induced by intra-plantar injection of CFA (100 μl, 1mg/ml *Mycobacterium*) in the left hind-paw. The contralateral hind-paw was injected with an equal volume of saline.

The CFA-induced hind-paw edema was evaluated by measuring the paw circumference after the behavioral tests at day 4, 7, 10, 14 and 21 post-injection. Rats were loosely immobilized in a towel, the hind-paw was held and a second experimenter measured the circumference by the use of a string. Three measures per paw and test day were performed and averaged. In order to normalize the paw size among rats and avoid weight and body size effects, the ratios of the circumferences of ipsi- to contralateral side were computed for each animal and expressed in percent.

### Blood sampling and measurement of plasma corticosterone levels

In order to evaluate the impact of MS and SS as well as their combination we determined the blood CORT concentration before, during and after the social stress period. Blood was taken from awake rats in the morning, between 8 and 9 am. To reduce stress reactions, animals were loosely held in a towel and a small incision in the distal part of the tail was performed by the use of a scalpel as described by Fluttert and colleagues [31]. About 300 μl of venous blood was collected with an EDTA-coated capillary tube (Microvette CB300). The blood samples were immediately placed on ice and then centrifuged at 12000 rpm for 10 min at 4°C. The segregated plasma was collected in 0.5 ml tubes and stored at -20°C until further analysis.

Plasma CORT levels were measured using an ELISA (Enzyme-Linked Immunosorbent Assay) kit (Assay Design, ADI-901-097). Data were acquired using a Sunrise Magellan plate reader and analyzed with the corresponding software package (Magellan Software, v6, 4 standard).

### Tissue sampling and RNA extraction

Following 21 days of peripheral inflammation animals were deeply anesthetized with isoflurane and decapitated. Spinal cord levels L5-L6 were removed and divided into ipsi- and contra-lateral side. Total RNA was extracted by the acid guanidium–thiocyanate–phenol–chloroform method using TRIzol® reagent (Life Technologies). After centrifugation, the aqueous phase was collected and the RNA was precipitated with isopropanol. The pellet was rinsed with 70% ethanol, air dried, dissolved in RNase free water and finally stored at -80°C until further analysis. RNA quality was assessed with the Experion Automated Electrophoresis Station (Bio-Rad) using StdSens chips (Bio-Rad). RNA concentration was measured by using the Nanodrop 2000 spectrophotometer quantification system (Isogen Life Sciences).

### Reverse transcription and Real-Time qPCR

Using the Improm-II reverse transcription kit (Promega), 500 ng of total RNA was reverse transcribed into cDNA with 0.5 μg/ml of oligo dT15 primer in a C1000 Touch thermocycler (Bio-Rad).

Real-Time qPCR experiments were performed on a CFX 96 real time system (Bio-Rad) with 12.5 ng of cDNA in a final volume of 20 μl by the use of PerfeCTa® SYBR® Green SuperMix (VWR) containing 2X reaction buffer with optimized concentrations of MgCl_2_, dNTPs, AccuStart Taq Polymerase, SYBR Green I dye, stabilizers and forward and reverse primers at 2 μM (for list of primers see Table 1).

**Table 1:** Primer sequences used in this study.

Name (gene)	Accession	Sequence	Amplicon size (bp)
Actin, beta	NM_031144.3	F: 5' GCT GAG AGG GAA ATC GTG CGT GAC 3'	96
		R: 5' GGA GGA AGA GGA TGC GGC AGT GG 3'	
GAPDH	NM_017008.4	F: 5' TCG GTG TGA ACG GAT TTG 3'	142
		R: 5' TGG GTA GAG TCA TAC TGG AA 3'	
GR (NR3C1)	NM_012576.2	F: 5' TGG AAA CCT GCT CTG CTT TG 3'	102
		R: 5' GAG GAG ACA AAC AGC ATG TG 3'	
NR1 (GRIN1)	NM_001270608.1	F: 5' GGT TGC GTG GGC AAC ACC AA 3'	80
		R: 5' CCG TCC GCA TAC TTA GAA GA 3'	
NR2a (GRIN2a)	NM_012573.3	F: 5' CAG ATA ACA ATA AGA ACC ACA AG 3'	83
		R: 5' AAC ATC GCT ACA GTC CTT 3'	
mGluR1 (GRM1)	NM_001114330.1	F: 5' CCT CTG CCA CCC CAT CTG AC 3'	84
		R: 5' GAG AGG AGG AGG CAA GCC CTT 3'	
mGluR5 (GRM5)	NM_017012.1	F: 5' ACA ACC TCT ACA GTG GTA CG 3'	147
		R: 5' GGC CCA AGT CAC AGA TTT TC 3'	
GFAP	NM_017009.2	F: 5' TAC AGG AAA TTG CTG GAG GG 3'	104
		R: 5' GAC ACA GAT TTG GTG TCC AG 3'	
Iba1 (AIF 1)	NM_017196.3	F: 5' AAT GAT GCT GGG CAA GAG AT 3'	129
		R: 5' ACC TCC AAT TAG GGC AAC TC 3'	
IL-1β	NM_031512.2	F: 5' AGA GTG TGG ATC CCA AAC AA 3'	105
		R: 5' GGA ACT GTG CAG ACT CAA AC 3'	
IL-6	NM_012589.2	F: 5' CCA GAG TCA TTC AGA GCA ATA C 3'	116
		R: 5' CTT CTC CAT TAG GAG AGC AT 3'	
TNF-α	NM_012675.3	F: 5' CCA GAG TCA TTC AGA GCA ATA C 3'	116
		R: 5' CTT CTC CAT TAG GAG AGC AT 3'	
GDNF	NM_019139.1	F: 5' GTG TTG CTC CAC ACC GCG TCT 3'	73
		R: 5' GGT CTT CGG CGG GCG CTT C 3'	
NGF		NM_001277055.1		F: 5' CAC GGA CAT CAA GGG CAA GGA 3'		96
				R: 5' GCT CGG CAC TTG GTC TCA AA 3'		

Primers were designed with the Beacon Designer™ software. Their sequence specificity was tested using the Basic Local Alignment Search Tool at NCBI and they were validated on spinal cord samples. The following steps were performed: polymerase activation at 95°C for 3 min, 40 cycles of amplification at 95°C for 10 sec, annealing at 61°C for 30 sec, recording of melting curves between 65°C and 95°C in 0.5°C intervals. All samples were run in triplicate and no-template controls were added as negative controls. Relative expression levels were estimated using the ΔΔCt-method with β-actin as the reference. Threshold cycle values (Cq) were used to calculate the amount of target gene mRNA in relation to the reference gene mRNA (β-actin). Therefore, ΔCq represents the difference between the number of cycles that were necessary to detect the PCR products of the target and the reference genes. ΔΔCq indicates the difference between the ΔCq of the experimental groups (CON+SS, MS and MS+SS) and the ΔCq of the control animals (CON). The data were expressed as 2^-ΔΔCq^ and the mean of the left (ipsilateral) inflamed side was computed for each group.

### Statistical analysis

Data are presented as mean ± SEM. Normality and homogeneity of variance were tested using Shapiro-Wilk and Levene’s tests respectively. The sucrose preference, CORT level and hot plate data were analyzed by a Kruskal-Wallis test followed by a Dunn’s multiple comparison test. For the forced swim test we used a one-way analysis of variance (ANOVA) and a Tukey’s multiple comparison *post hoc* test to check for differences between groups. The statistical analyses for the paw circumference measurements and the von Frey experiments were carried out using a two-way (time x condition) repeated measures ANOVA followed by Tukey’s multiple comparison *post hoc* test.

For all gene expression comparisons, the ipsilateral spinal cord of CON served as reference and the relative expression level was set to 1. The expression levels of the treatment groups MS, SS and MS+SS were expressed as fold of CON. For statistical analysis, these relative expression levels (fold) were compared using the one-way ANOVA followed by Tukey’s multiple comparison *post hoc* test.

## Results

### Behavioral assessments

#### Stress manipulations did not affect sucrose preference

**Fig 2:**
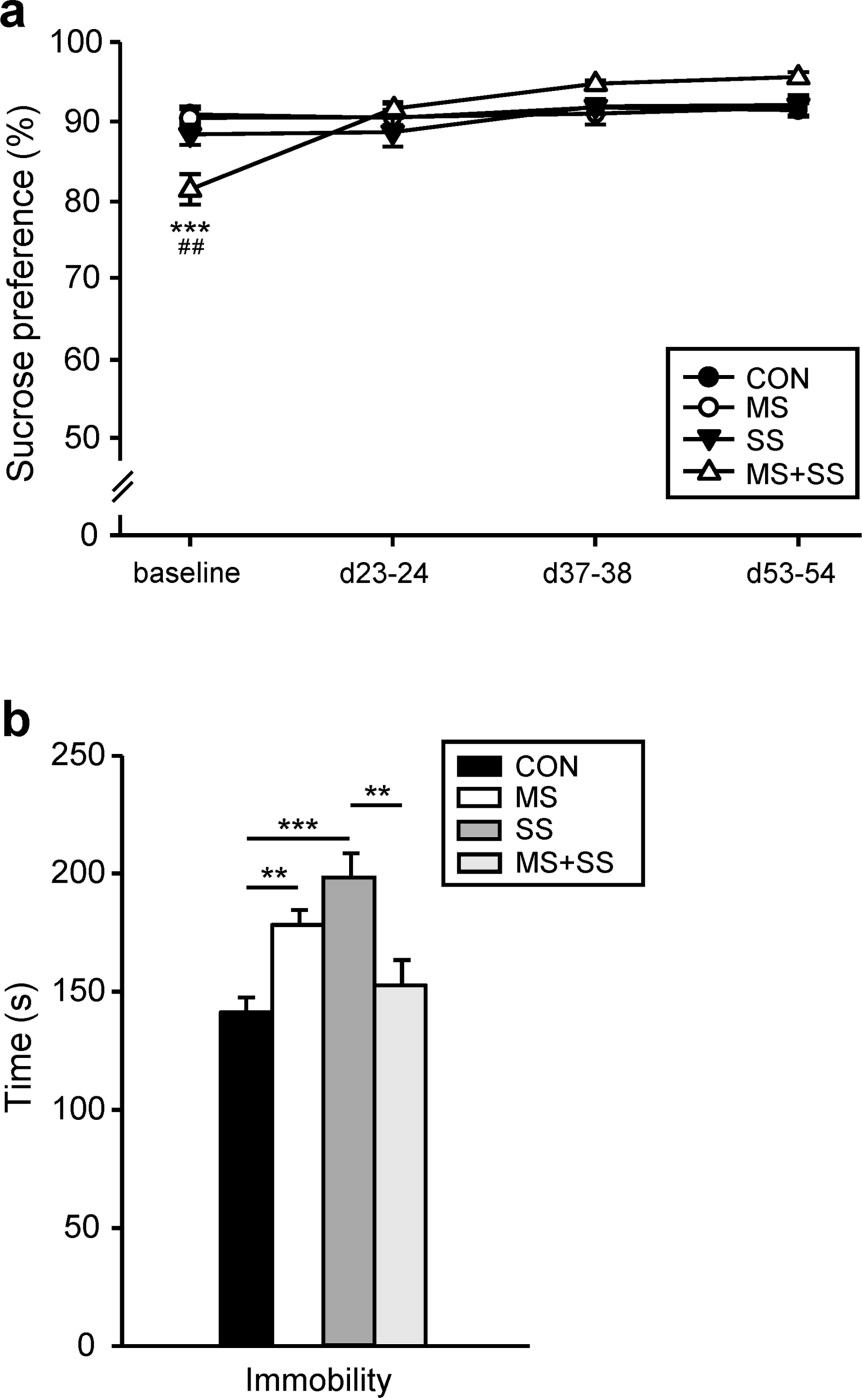
Depression-like behavior. (a) Sucrose preference was measured before (baseline), during (d23-24 and d37-38) and after (d53-54) the social stress period. The four groups, control (CON, black circles, n = 23), animals exposed to maternal separation (MS, white circles, n = 16), rats exposed to social stress (SS, black triangles, n = 12) and the combined maternal separation and social stress subjected group (MS+SS, white triangles, n = 12) exhibited similar preference levels throughout the stress period. At baseline the MS+SS group unexpectedly showed significantly lower sucrose preference, although it cannot be interpreted as anhedonic behavior. Data are expressed as mean ± SEM. * represents a significant difference between CON and MS+SS (***p < 0.001). ^#^ indicates a significant difference between MS and MS+SS (^##^p <0.01). (b) Forced swim test: the time (s) rats spent immobile was measured after the social stress period at d54-55. MS (white bar) and SS (dark gray bar) animals exhibit a significant higher immobility than CON (dark bar) whereas the combination of the two stressors (MS+SS, light grey bar) reduced the immobility time to control levels. Data are expressed as mean ± SEM. (**p<0.01, ***p<0.001).

Under baseline conditions, the group that was assigned to MS+SS conditions unexpectedly showed a significantly lower sucrose preference as compared to CON (p<0.001) and to MS (p<0.01) (Fig 2A). This result can however not be interpreted as anhedonic behavior since the commonly used criterion of an at least 65% reduction was not reached [32,33]. Over the course of the experiment, no other differences between groups were observed in this respect.

#### MS, SS, but not their combination altered FST-related behavior

The time (s) rats stayed immobile during the testing session significantly depended on the different manipulations (F_3, 59_ = 10.168, p<0.001) (Fig 2B). The *post hoc* analysis revealed a significant increase of immobility in the MS (178.21 ± 6.42 s; p<0.01) and SS (198.30 ± 10.37 s; p<0.001) groups when compared to CON (141 ± 6.3 s). Combining the two stressors, MS+SS, did not result in a further increase of immobility but, in contrast, reduced the time spent immobile to control levels (152.63 ± 10.79 s).

#### Impact of the various stressors on mechanical thresholds

**Fig 3:**
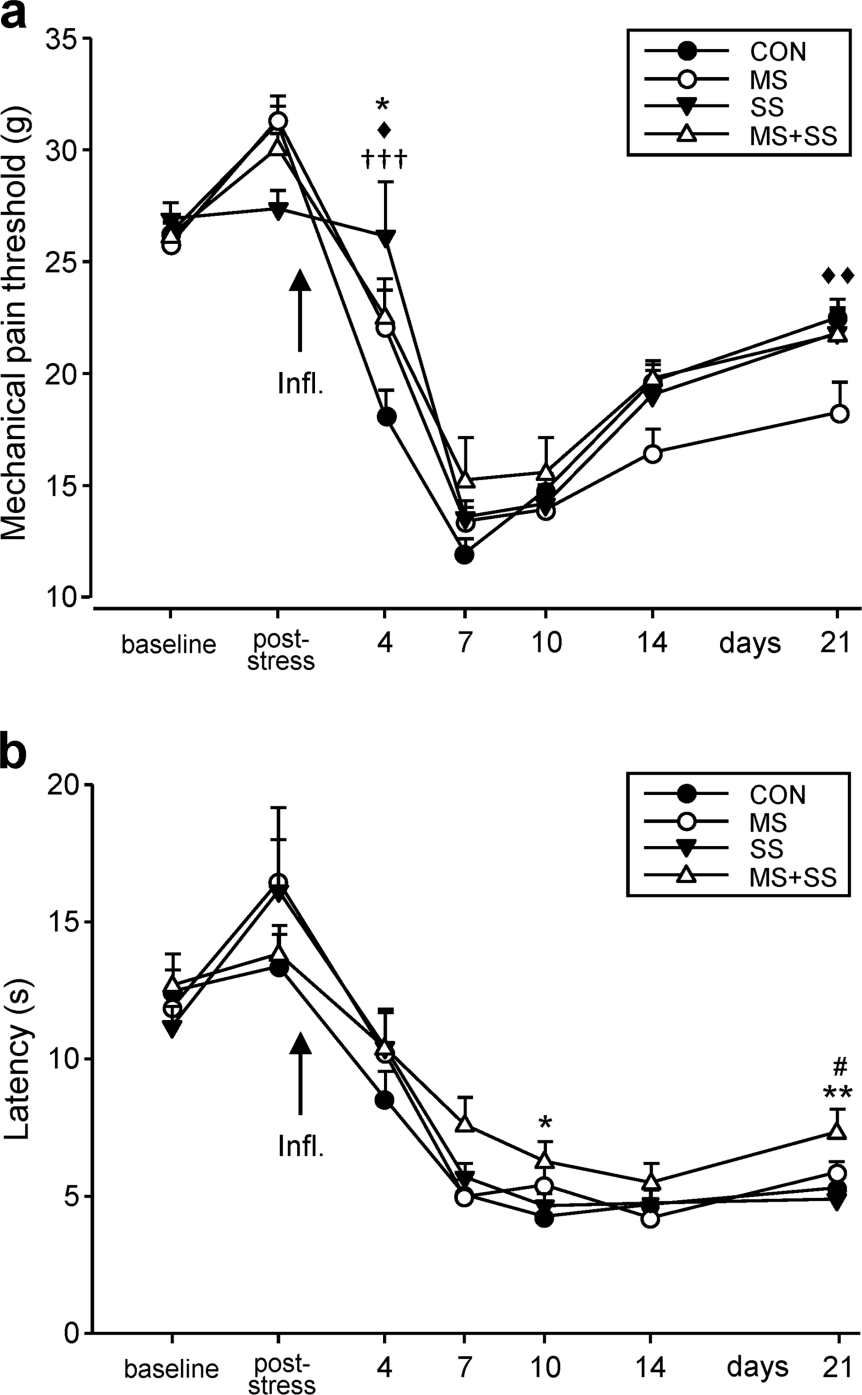
Effect of early life stress (MS) and social stress (SS) on inflammation-induced mechanical and thermal allodynia/hyperalgesia. (a) Mechanical pain thresholds were assessed before (baseline) and after the social stress period (post-stress) in all four conditions (CON: black circles, n = 23, MS: white circles, n = 16, SS: black triangles, n = 12, MS+SS: white triangles, n = 12). CFA-induced inflammation of the left hind-paw resulted in a decrease of pain thresholds in all groups, peaking at day 7 and partially recovering until the end of the experiment at day 21. The onset of mechanical allodynia/hyperalgesia was significantly delayed in all three stress groups (MS, SS, MS+SS) at day 4 when compared to CON. The recovery (day 21) was only significantly delayed in MS animals. Data are expressed as mean ± SEM. * represents a significant difference between CON and MS+SS (*p < 0.05), ^♦^ between CON and MS (^♦^p <0.05, ^♦♦^p <0.01) and ^**†**^ between CON and SS (^**†††**^p <0.001). (b) Thermal pain thresholds did not differ between groups (CON: black circles, MS: white circles, SS: black triangles, MS+SS: white triangles) before (baseline) and after the social stress period (post-stress). Injection of CFA into the left hind-paw induced thermal hyperalgesia in all groups persisting until the end of the experiment at day 21. MS+SS animals showed less hyperalgesia than CON at day 10 and less when compared to CON and MS at day 21. Data are expressed as mean ± SEM. * represents a significant difference between CON and MS+SS (*p < 0.05, **p<0.01) and ^#^ between MS and MS+SS (^#^p <0.05).

Mechanical pain thresholds did not differ between groups at baseline (Fig 3A). Following social stress, none of the groups displayed significantly altered mechanical thresholds. After CFA injection animals progressively developed tactile allodynia/hyperalgesia peaking at day 7 and then slowly recovering without coming back to baseline levels at day 21. At day 4, inter-group comparisons revealed a significantly lower mechanical threshold of CON when compared to MS (p<0.05), SS (p< 0.001) and MS+SS (p<0.05). From day 7 to 14, no differences between groups were observed and at day 21 MS had a significantly lower mechanical threshold than CON (p<0.01), pointing to a slower recovery of the maternally separated animals.

#### Effect of early life (MS) and social stress (SS) on thermal hyperalgesia

In the hot plate test, no significant difference in noxious heat sensitivity was found between any of the groups at baseline or following the social stress period (Fig 3B). The induction of inflammation rapidly and lastingly decreased the latency for the animals to present nocifensive behaviors. At day 10 post-injection, MS+SS animals presented a significantly lower thermal sensitivity than CON (p<0.05). No significant difference between any of the groups was seen 14 days after CFA injection but the MS+SS group showed a significant reduction of thermal sensitivity when compared to CON (p<0.01) and MS (p<0.05) 21 days after CFA injection.

#### Evaluation of CFA-induced paw edema

**Fig 4:**
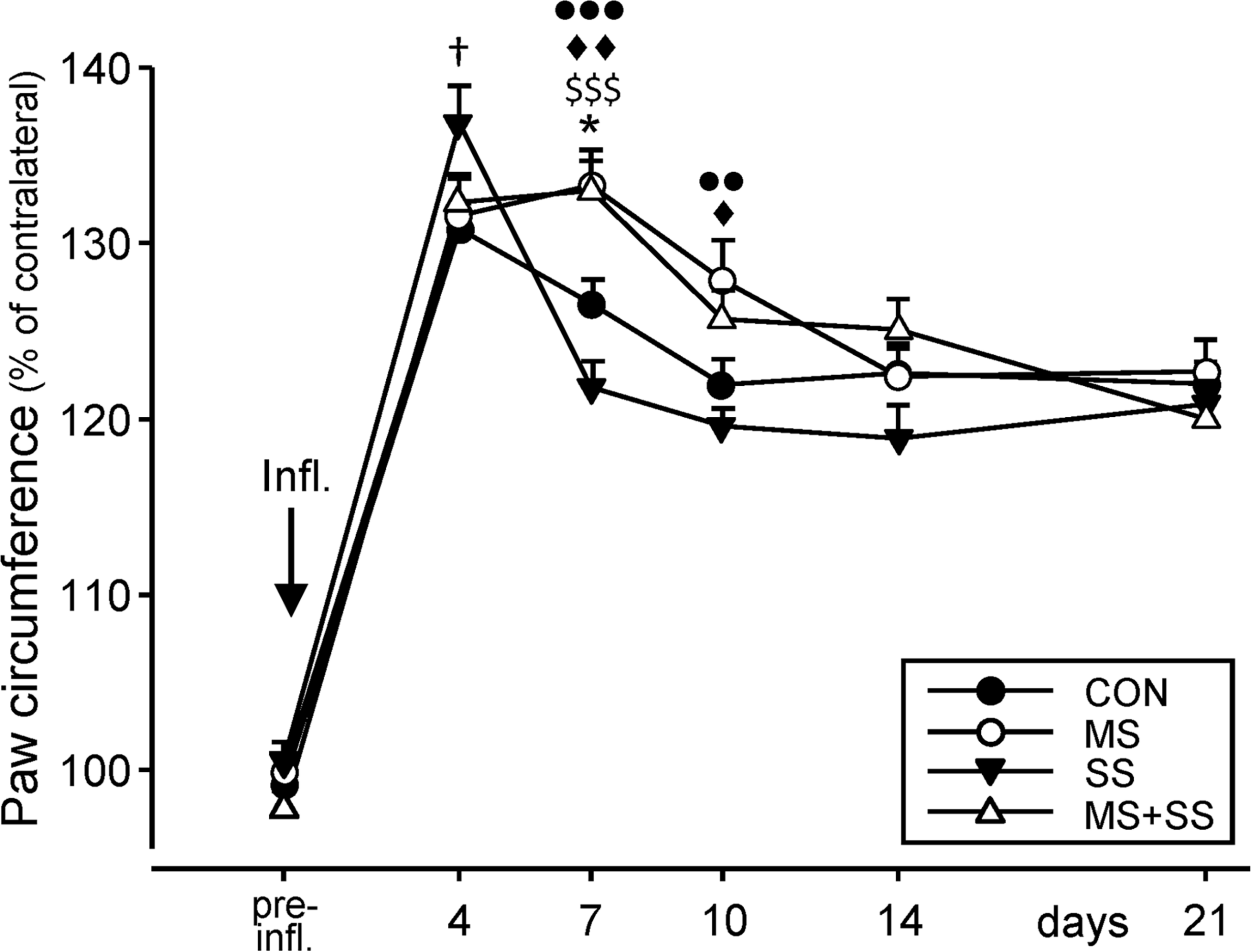
Development of inflammation-induced edema. The circumference of the left hind-paw (expressed as % of the circumference of the right hind-paw) was assessed before the induction of inflammation (pre-infl.). No difference was obtained between the four groups (CON: black circles, n = 23, MS: white circles, n = 16, SS: black triangles, n = 12, MS+SS: white triangles, n = 12). CFA injection produced a pronounced swelling which peaked at day 4 in the CON and SS groups and at day 7 in MS and MS+SS leading to significant differences in the early phase of edema development between CON and SS (^**†**^*p*<0.05) at day 4, between CON and MS (^♦♦^*p*<0.01), CON and MS+SS (**p<0.05), SS and MS+SS (^$ $ $^*p*<0.001), MS and SS (^●●●^*p*<0.001) at day 7 and between CON and MS (^♦^*p*<0.05) and MS and SS (^●●^*p*<0.01) at day 10. Data are expressed as mean ± SEM.

No significant differences in paw circumference (data computed as percentage of the contralateral, non-injected hind-paw) were found between any of the 4 groups before the induction of inflammation (Fig 4). CFA injection produced a significant swelling of the paw in every group (p<0.001 for all time points) as revealed by a two-way repeated measures ANOVA (F_5, 348_ = 194.1, p <0.0001). A significant group effect (F_3, 348_ = 5.045, p = 0.002) and interaction (F_15, 348_ = 2.948, p<0.001) were also seen. In CON (130.8 ± 0.83%) and SS (136.8 ± 2.17%; p<0.05 vs. CON) the paw circumference peaked on day 4 while in MS (133.3 ± 2.01%) and MS+SS (132.9 ± 1.70%) it peaked on day 7 post-inflammation, pointing to a small delay in the development of edema in MS animals. At this later time point, significant differences between groups were observed between CON and MS (p<0.01), CON and MS+SS (p<0.05), MS and SS (p<0.001), and finally MS+SS and SS (p<0.001). At day 10, the edema also decreased in the MS and MS+SS groups. Significant differences could however still be seen between MS and CON (p<0.05) and between MS and SS (p<0.01). On later days the paw circumferences leveled off to about 120% in all four groups.

#### Measurement of blood CORT levels

**Fig 5:**
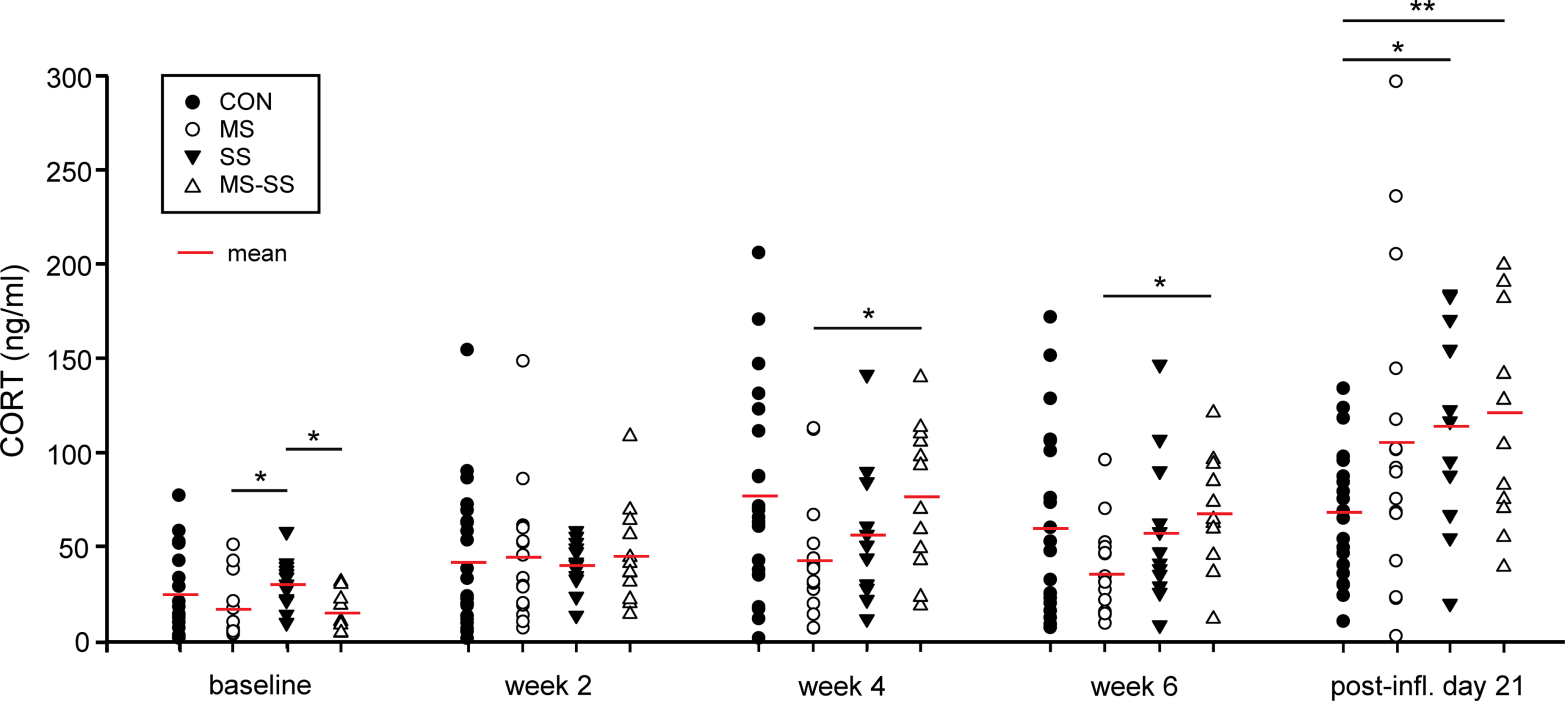
Effect of early life (MS) and social stress (SS) and their combination (MS+SS) on blood serum corticosterone levels. Blood was taken and corticosterone levels were measured before (baseline), during (end of week 2 and 4) and after (week 6) the social stress period as well as 21 days after the onset of inflammation (post-infl). At baseline, the maternally separated animals (MS, white circle, n = 16 and MS+SS, white triangles, n = 12) had lower blood corticosterone concentrations when compared to SS (black triangles, n = 12) but surprisingly not in comparison with controls (CON, black circles, n = 23). Two weeks after the start of social stress in the respective groups, the corticosterone levels increased in all animals, independent of the stress condition. After week 4, the corticosterone concentration further increased in CON, SS and MS+SS but not in MS and roughly stayed at that level after week 6. The CFA-induced inflammation increased the concentration of serum corticosterone as well as the intra-group variability in the stress groups MS, SS and MS+SS but not in CON. Data are expressed as mean ± SEM. (*p<0.05, **p<0.01).

Baseline measurements revealed significantly reduced corticosterone levels in maternally separated animals (MS and MS+SS groups) (Fig 5) when compared to SS (p<0.05) but surprisingly not to CON animals although CON and SS groups where not subjected to different manipulations at this time point. Two weeks after the start of the social stress paradigm, the CORT levels increased in all experimental groups, but no inter-group differences could be seen. In CON and MS+SS the ongoing stress resulted in a further increase of the blood CORT concentration 4 weeks after the beginning of the social stress period, leading to a significant difference between the MS and MS+SS groups (p<0.05). After the 6^th^ week of social stress, no further changes were observed. Finally, after 21 days of inflammation CON animals had similar CORT levels as before inflammation. However, in the groups with a history of stress manipulation, the blood CORT concentration was likewise increased but significant differences were only revealed between SS vs. CON (p<0.05) and MS+SS vs. CON (p<0.01).

### Biochemical assessments

#### Stress-mediated alterations of spinal glucocorticoid and glutamatergic receptors mRNA expression

**Fig 6:**
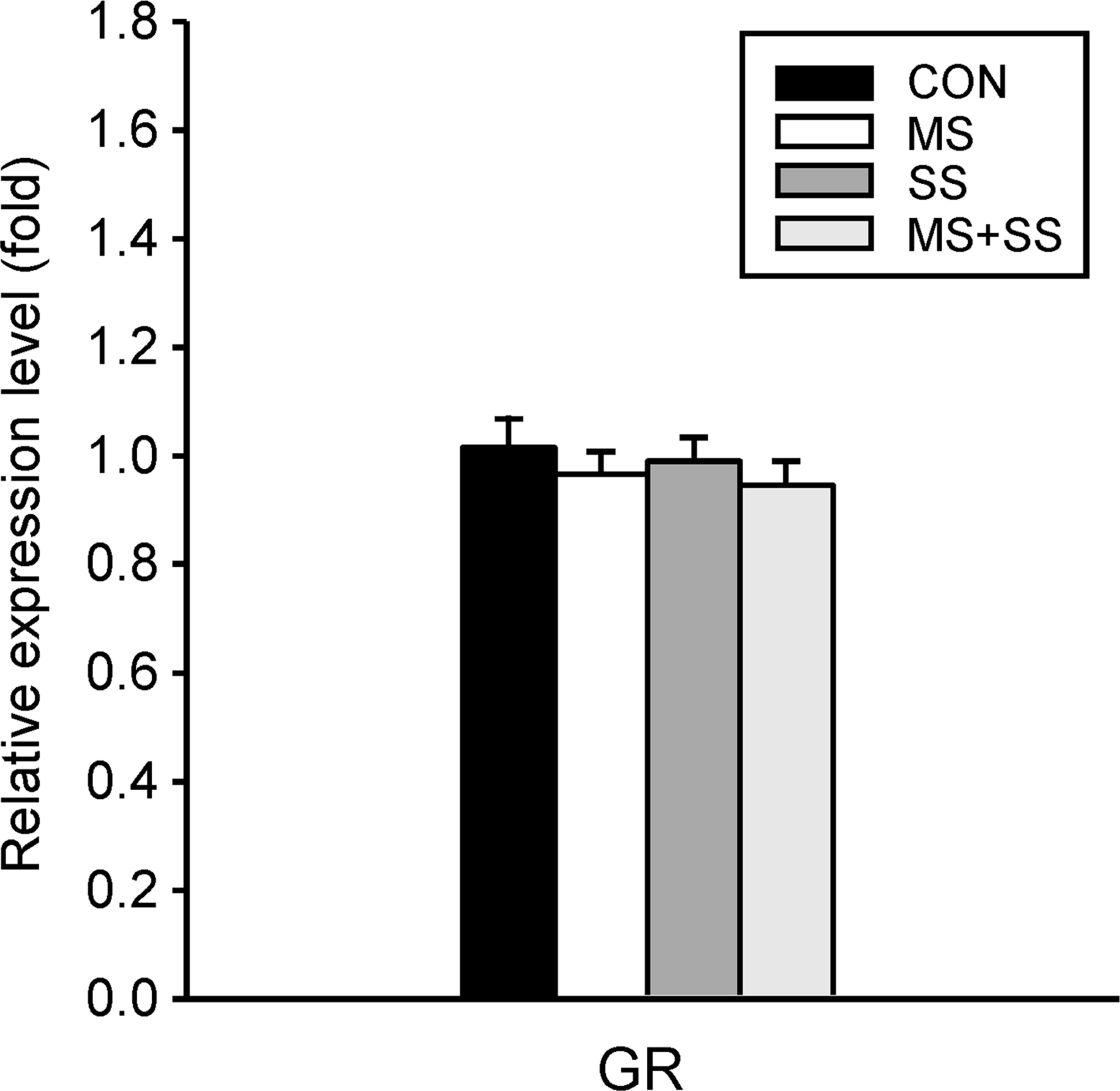
Regulation of the spinal glucocorticoid receptor (GR). Gene expression of GR was examined in the ipsilateral (left) spinal cord segment L5/L6 of controls (CON, black bars, n = 23), maternally separated rats (MS, white bars, n = 16), animals subjected to social stress (SS, dark grey bars, n = 12) and rats that were exposed to both stressors (MS+SS, light grey bars, n = 12), 21 days after induction of inflammation. GR mRNA expression was not influenced by any of the three stress conditions. Data are expressed as relative expression level (fold) of CON (control group = 1) and are shown as mean ± SEM.

*Glucocorticoid receptor (GR)*: The regulation of the GR mRNA expression was not affected by any of the stress conditions (Fig 6).

**Fig 7:**
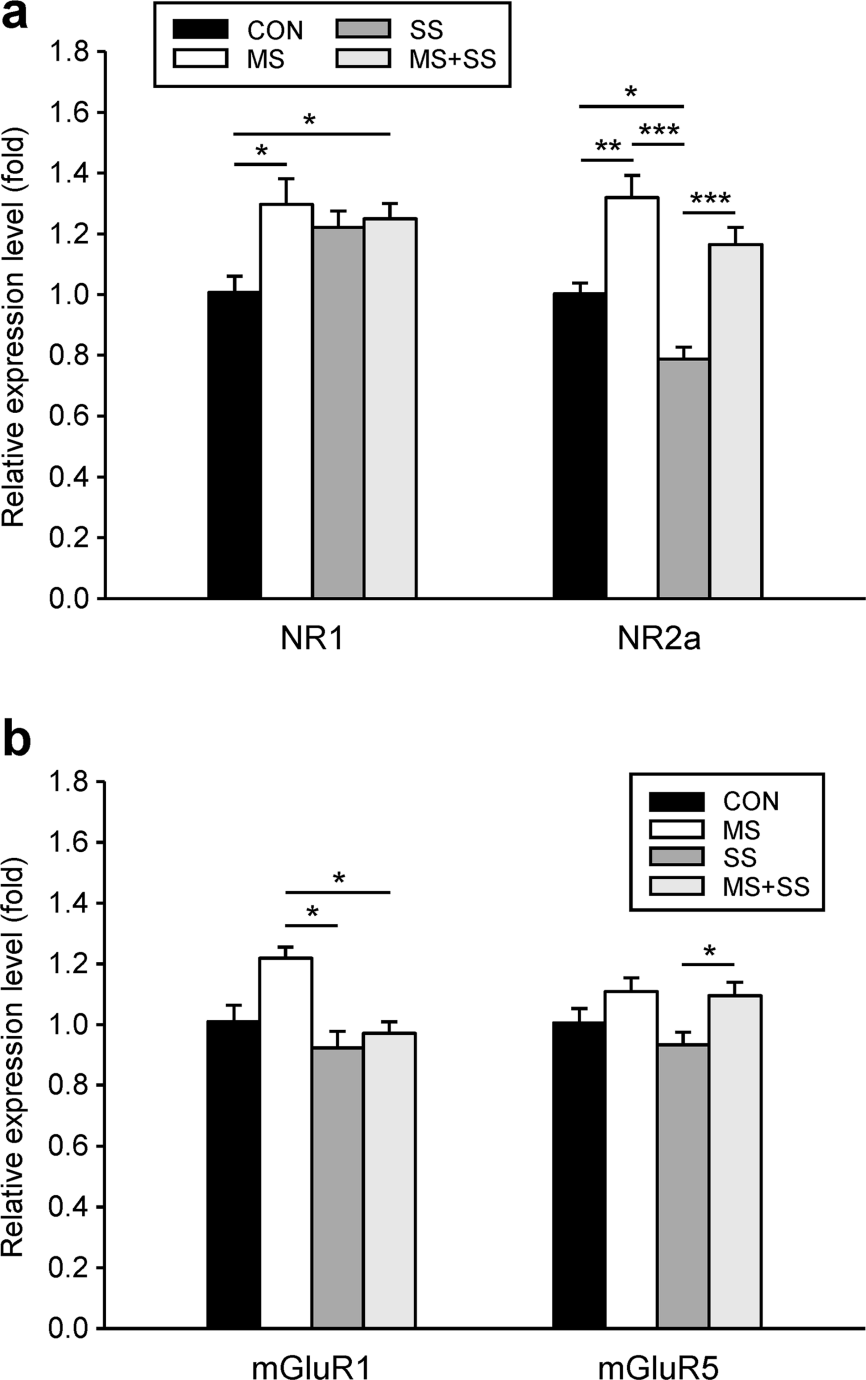
Regulation of spinal ionotrophic and metabotrophic glutamate receptors. The gene expression of two subunits of the ionotrophic NMDA receptor, NR1 and NR2a (a), and of the group I metabotropic receptors mGluR1 and mGluR5 (b) was examined in the ipsilateral spinal cord segment L5/L6 of controls (CON, black bars, n = 23), maternally separated rats (MS, white bars, n = 16), animals subjected to social stress (SS, dark grey bars, n = 12) and rats that were exposed to both stressors (MS+SS, light grey bars, n = 12), 21 days after induction of inflammation. Data are expressed as relative expression level (fold) of CON (control group = 1) and are shown as mean ± SEM. (a) left: NR1 mRNA was slightly upregulated in all three stress groups reaching significance in MS and MS+SS animals. (a) right: NR2a mRNA was upregulated in MS, downregulated in SS and not significantly altered in MS+SS. (b) left: mGluR1 mRNA showed an upregulation trend in the MS group. SS and MS+SS conditions had no influence on the mGluR1 mRNA expression. **(**b) right: No significant alteration of the mGluR5 mRNA level was found in the three stress conditions when compared to CON. (*p<0.05, **p<0.01, ***p<0.001).

*Glutamatergic system*: After 21 days of inflammation, the NR1 mRNA levels were upregulated in all the experimental groups as compared to CON (1.01 ± 0.05) but reached statistical significance only in MS (1.30 ± 0.08, p<0.05) and MS+SS (1.25 ± 0.05, p<0.05) (Fig 7A). NR2a expression was differentially affected by stress conditions since it was upregulated in MS (1.32 ± 0.07, p<0.01), downregulated in SS (0.79 ± 0.04, p<0.05) and not significantly altered in MS+SS (1.17 ± 0.06) when compared to CON (Fig 7B).

Regarding mGLUR1, no stress group presented a significant difference to CON (Fig 7B) (1.22 ± 0.03). However, significant differences were found between MS and SS (0.92 ± 0.06, p<0.05) and MS+SS (0.97 ± 0.03, p<0.05). In the case of mGluR5 mRNA there was no significant alteration in the stress groups relative to CON. Only a significant inter-group difference could be found for SS vs. MS+SS (p<0.05) (Fig 7B).

#### Stress-induced modulations of immunocompetent cell marker and pro-inflammatory cytokines mRNA expression

**Fig 8:**
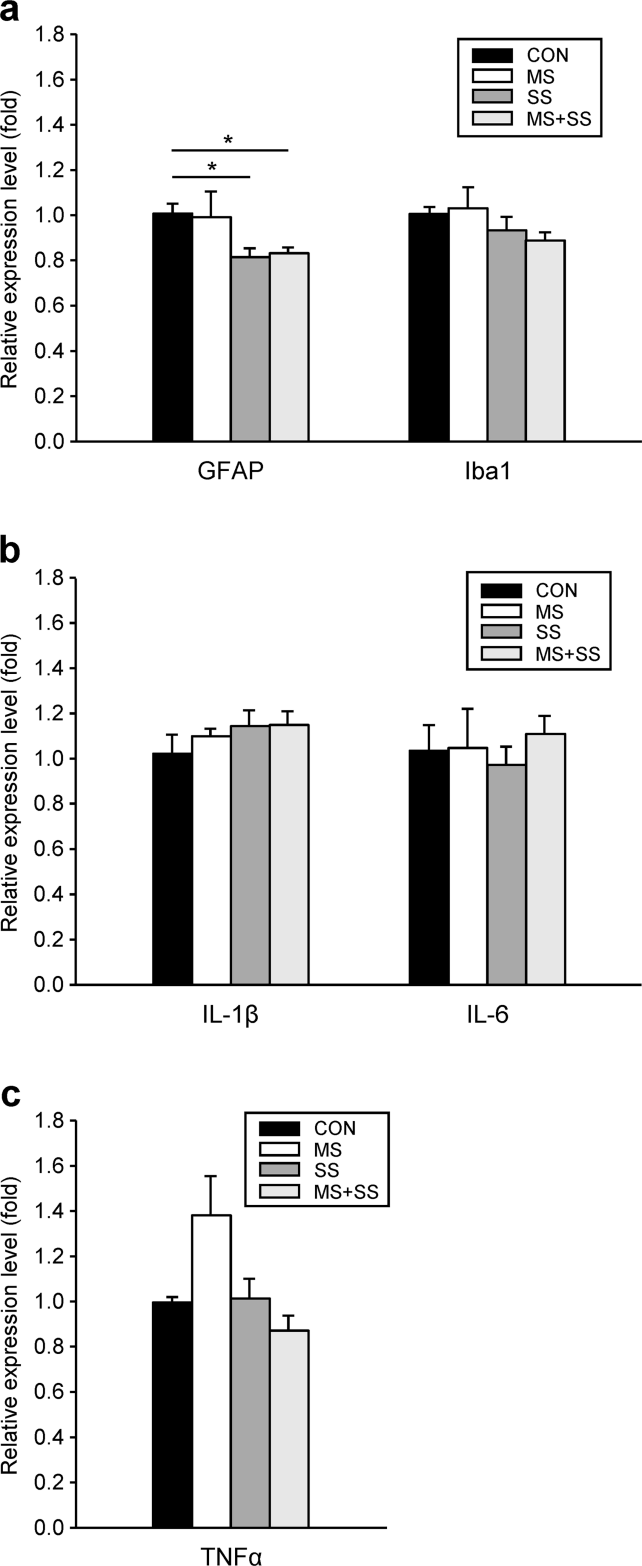
Regulation of glial activation markers and pro-inflammatory cytokines. The gene expression of the astrocyte marker GFAP and the microglia marker Iba1 (a), and of the pro-inflammatory cytokines IL-1β, IL-6 and TNFα (b, c) was examined in the ipsilateral spinal cord segment L5/L6 of controls (CON, black bars, n = 23), maternally separated rats (MS, white bars, n = 16), animals subjected to social stress (SS, dark grey bars, n = 12) and rats that were exposed to both stressors (MS+SS, light grey bars, n = 12), 21 days after induction of inflammation. Data are expressed as relative expression level (fold) of CON (control group = 1) and are shown as mean ± SEM. (a) left: GFAP mRNA was downregulated in SS and MS+SS animals, but not in the MS group (*p<0.05). (a) right: the three stress conditions (MS, SS, MS+SS) had no influence on Iba1 mRNA expression. (b): the mRNA expression of IL-1β (left) and IL-6 (right) was not altered by stress. (c): TNFα mRNA expression was slightly, but not significantly upregulated in MS. Social stress (SS) and the combination of early life and social stress (MS+SS) had no effect on the regulation of the TNFα gene.

*Activation of astrocytes and microglia*: 21 days after CFA-induced hind-paw inflammation a significant reduction of GFAP mRNA expression level was observed in animals subjected to SS (0.81 ± 0.08, p<0.05) and MS+SS (0.83 ± 0.02, p<0.05) but not in MS (0.99 ± 0.11) when compared to CON (1.01 ± 0.04) (Fig 8A). No significant differences between groups were observed for the regulation of Iba1 mRNA expression (Fig 8A).

*Pro-inflammatory cytokines*: After 21 days of inflammation, no significant inter-group differences could be found in the mRNA expression levels of IL-1β, IL-6 or TNF-α. (Fig 8B).

#### Stress-mediated alterations in the mRNA expression of growth factors GDNF and NGF

**Fig 9:**
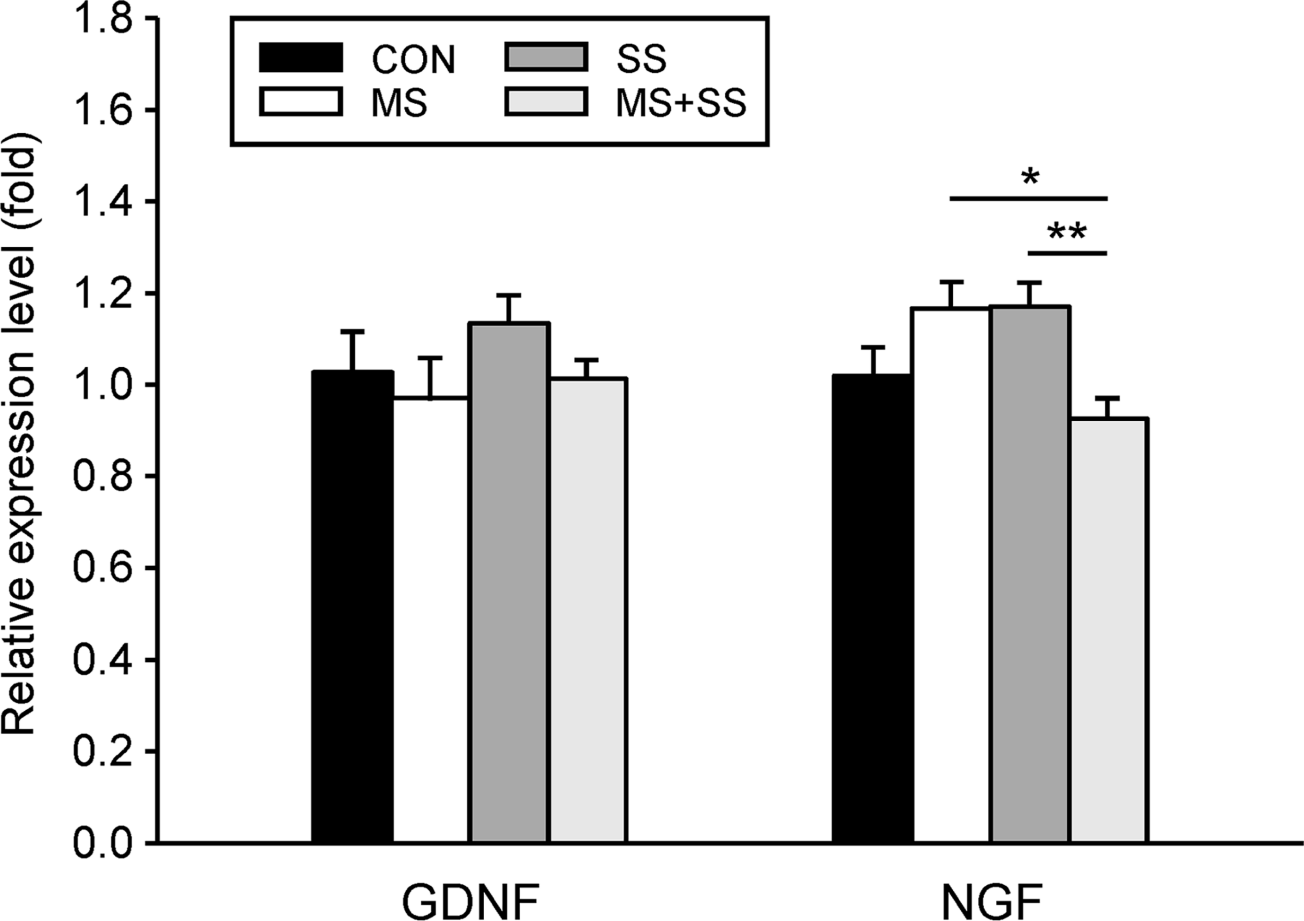
Regulation of spinal growth factors. The gene expression of the growth factors GDNF (left) and NGF (right) was examined in the ipsilateral spinal cord segment L5/L6 of controls (CON, black bars, n = 23), maternally separated rats (MS, white bars, n = 16), animals subjected to social stress (SS, dark grey bars, n = 12) and rats that were exposed to both stressors (MS+SS, light grey bars, n = 12), 21 days after induction of inflammation. Data are expressed as relative expression level (fold) of CON (control group = 1) and are shown as mean ± SEM. Left: the different stressors had no effect on the mRNA expression of GDNF. Right: None of the three stress conditions resulted in significant alterations of NGF mRNA expression when compared to the controls (CON). Slight upregulation in MS and SS animals and slight downregulation in the MS+SS group led to significant differences between MS and MS+SS and between SS and MS+SS. (*p<0.05, **p<0.01).

The three examined stress conditions did not differentially regulate spinal GDNF mRNA levels since no significant inter-group differences were obtained (Fig 9). Concerning the mRNA expression of NGF, none of the stress groups exhibited a significant difference to the CON group (1.02 ± 0.07). On the contrary, as NGF mRNA expression decreased in MS+SS animals (0.93 ± 0.04) it led to significant differences of this group to MS (p<0.05) and SS (p<0.01).

## Discussion

Considering the high comorbidity between stress-related disorders and chronic pain we sought to determine if individuals exposed to ELS and then undergoing a prolonged period of social stress during adulthood are rendered more susceptible to chronic inflammatory pain.

We previously studied the effects of MS, an established ELS paradigm on the development of neuropathic [10] and inflammatory pain [11] in adult age. While these studies revealed that MS may be beneficial, in the sense that it promotes a form of resilience against pain hypersensitivity, other studies showed that MS can lead to an enhanced vulnerability to chronic pain conditions [34,35].

### Impact of MS and/or SS on anxio-depressive-like disorders

Chronic stress paradigms commonly used in adult animals and basing on repeated exposure to adverse physical stimuli such as restraint do not readily mimic the etiology of stress-related disorders found in humans since the increased prevalence of psychiatric disorders is mainly related to adverse psycho-social conditions [27,36]. Other experimental approaches such as the one used in the present study, the resident intruder or the visible burrow system paradigms may hence be more relevant for the elucidation of coping strategies and vulnerability/resilience mechanisms [37].

We did not observe significant differences in sucrose preference between any of the groups after 2, 4 and 6 weeks of social stress exposure. Surprisingly however, MS+SS animals presented a significantly reduced sucrose preference compared to all the other groups at baseline. In spite of the significantly reduced sucrose preference of the MS+SS animals, they did not reach the reduction to 65% defined as the threshold value for anhedonia [32,33].

The forced swim test (FST) has long been considered as a paradigm testing behavioral despair, a core symptom of depression as it was traditionally used for screening potential antidepressant molecules [38]. However, more recent views claim that the FST provides a model for responses to an acute uncontrollable stress rather than for depression [39]. Our results show that animals undergoing MS or SS presented enhanced immobility compared to CON animals. Immobility is considered to reflect passive coping when facing an uncontrollable stress [40]. Previous experiments demonstrated that micro-injections of GABA, lidocaine or antidepressant in structures involved in the processing of stress and pain such as the amygdala [41] or the periaqueductal gray [42] led to a reduction of immobility time in the FST. Since these structures are involved in the affective dimension of pain [43], the enhanced immobility observed in MS and SS groups could be linked to an altered pain sensation or unpleasantness. Interestingly however, MS+SS animals displayed a total time of immobilization behavior comparable to the one displayed by the CON rats pointing to the possibility that the combination of MS and SS may have led to a better coping when facing stressful situations.

Taken together our sucrose preference and FST-related results may seem confusing since anhedonia has been associated with a decreased immobility in the FST [44]. However other investigators showed that these behaviors could be dissociated [45]. Moreover, it has been emphasized that anhedonia and passive coping following a chronic stress were linked to submissive behavior [46]. We did not test submission in our experiment. However, we believe that the social instability model we used here, mingling cage mates and consequently also dominance/submission relationships is hardly reconcilable with tests of susceptibility to submission because individuals were not put in a situation where they are forcefully dominated or dominating. Indeed, in our SS paradigm a rat displaying submissive behavior on one week might be dominant on the next week when the composition of the cage partners is changed.

### Stress-induced modification of CORT levels

CORT was measured to assess possible ELS-induced changes in hypothalamic-pituitary-adrenal (HPA) axis activation during SS and at the end of the chronic inflammatory period. ELS has been shown to lead to higher CORT levels in later life but this is not a uniform finding since factors like genetic background, nature of ELS and later experiences can differentially influence the HPA axis [1,2]. In our hands, MS did not significantly impact CORT levels as shown in the baseline measurement. It is important to note that the baseline measurements were done at the end of the late rodent adolescence period (P46 to P59) [47]. At this age the HPA axis is still sensitive [48,49] to maturation-related phenomena such as puberty that could influence CORT levels in MS animals [50]. The lack of difference between the CON and SS groups before and throughout the SS period is surprising but could be related to the timing of the SS. Indeed, SS performed during adolescence has been shown to cause lasting alterations of behavioral [51] and neuroendocrine [27] features. However, in our experiments rats underwent SS during the late adolescence/beginning of adulthood, thus possibly after the high adolescence-related vulnerability window and consequently may have displayed lower alterations of their HPA axis. Early experiments have focused on SS in adult rats, however only a confrontation with unfamiliar and aggressive males in combination with the presence of females provoked social instability and was linked to increased adrenal weight and CORT levels [29]. The fact that we did neither assess our animals for dominant behaviors nor introduce a competition for females could explain the lack of elevated CORT levels. After 21 days of inflammation we observed a significant increase of CORT in SS and MS+SS groups and a strong tendency in MS when compared to the CON group. This could suggest that the CFA-induced increase in CORT levels may have been potentiated by a history of SS but not MS. CORT levels have been shown to increase with the onset of rheumatoid arthritis triggered by CFA injection [52] but results are not always congruent [53]. In this context it should be kept in mind that CFA-induced arthritis aggravating factors may depend upon aspects like rat gender and animal supplier [54,55] that may in turn explain a lack of CORT increase in some animals. Since the loss of CORT diurnal rhythm has also been reported following CFA injection [52], measures done prior to and after 21 days of inflammation may be difficult to compare. Nevertheless, our result seems to indicate that social stress leads to an enhanced HPA axis activity during a chronic inflammatory state. Indeed, despite a trend toward an increase observed in MS, only SS and the combination of MS+SS led to significant differences. However, the high level of variability of our ELISA data precludes a confirmation of a heightened HPA axis activity during chronic inflammation in the MS group.

The possible sustained activation of the HPA axis as indicated by elevated CORT levels may modulate immune responses since it leads to alterations in the release of pro- and anti-inflammatory cytokines [56]. On days 4 and 7 after CFA-induced inflammation, we observed that MS animals presented the most pronounced edema. However, despite comparable levels of CORT in CON, SS and MS+SS groups at the end of the SS period, CFA-induced paw edemas differed. The development of inflammation depends on the clinical and preclinical state [54] of the immune system which can be influenced by environmental factors such as stress. As our study only evaluated biochemical markers during inflammation, it is premature to speculate on the direct link between circulating CORT and CFA-induced paw edema.

### Chronic stress related modulation of mechanical and thermal CFA-induced hyperalgesia

The present study confirms prior results [10,51] as we do not see differences in thermal and mechanical sensitivity between any of the groups before inflammation. However the measurement of noxious sensitivity during periods of ongoing inflammation may generate misleading results. CFA-induced inflammation has been divided in different early and late phases [18,57] and animals with diverging stress experiences may display differential reactivity at these different stages. In our study, all stress groups displayed lower mechanical but similar thermal sensitivity as compared to CON during the early or subacute phase of CFA-induced inflammation. While acute stress is commonly claimed to induce “stress-induced analgesia” (SIA) [58], the situation is less clear for chronic stress. It has e.g. been shown that MS or SS may either induce analgesia or hyperalgesia [59].

Interestingly, the MS group displayed decreased mechanical thresholds at day 21 after CFA injection. A similar shift from analgesia in the early phase to hyperalgesia in the late phase was previously observed around day 21 [51]. In that particular study, SS rats displayed an enhanced cold allodynia at day 28 after chronic constriction injury (CCI) while being less sensitive shortly after CCI. A comparable phenomenon was observed in MS animals undergoing CCI since their mechanical sensitivity kept decreasing until post-CCI day 21 whereas CON rats started to recover [10]. Longer observation periods may be required to confirm that these results indicate a shift from analgesia to hyperalgesia, possibly due to a general allostatic load [59,60].

Noxious thermal sensitivity did not present the same pattern of modulation by stress as seen for the mechanical sensitivity. Indeed, despite CFA eliciting the expected decrease of paw withdrawal latency (PWL), no significant differences between any of the groups were seen during the early phase of the inflammation. In order to understand how and why the stress seems to impact the noxious modalities in a differential way, further experiments are needed. Also, it is important to consider other levels of regulation. For instance, Banik and colleagues [61] investigated the latency to develop CFA-induced paw edema in different rat strains. Interestingly, Sprague Dawley rats turned out to be the less sensitive to CFA even following a large dose of CFA (1.2mg). This was indicated by a longer latency or even reduced occurrence of the development of arthritis, accompanied by a greater paw volume variation and less pronounced edema. In the present study we did not observe a high variability regarding the paw swelling. Interindividual differences in susceptibility and/or the longer latency of arthritis establishment together with the enhanced variability inherent to the multiple manipulations could have led to mitigated results. Furthermore, other investigators have observed a link between blood and paw tissue content of pro-inflammatory cytokines [61,62] and susceptibility to inflammation and inflammatory pain. Indeed, interleukins can activate the transient receptors potential cation channel subfamily V (TRPVs) [63] and differences in interleukin levels displayed by more or less susceptible animals could lead to differential heat sensitivity.

### Impact of CFA-induced inflammation on spinal GR expression

Spinal GR expression was previously studied in the context of neuropathic pain [13] and GR upregulation seemed to be required for spinal sensitization [12,13,64]. To our knowledge however, similar studies were not conducted for persistent inflammatory conditions. In our hands, GR mRNA expression did not differ between all four groups at day 21 of inflammation. This result is in agreement with previous studies from our laboratory where we did not observe any change of spinal GR expression in animals undergoing CCI, MS or the combination of both [10].

### Modulation of glutamatergic receptors by stress in an inflammatory context

Accumulating evidence shows that chronic stress and glucocorticoid levels have a significant effect on glutamatergic synapses [65]. NMDA receptors are a crucial component for the establishment and maintenance of central sensitization involved in chronic pain [66]. However, alterations in the expression of NMDA subunits vary across brain structures and depend on stress parameters [67]. Our data indicate that the spinal cord mRNA content of the NR1 subunit of the NMDA receptor was equivalent after 21 days of inflammation in all three stress groups. As NR1 is the constitutive subunit of the NMDA receptor [68] this result might hint at an enhanced spinal sensitization and higher noxious sensitivity in stressed animals. However, the properties of NMDA receptors are largely defined by their other subunit composition [69]. NR2a containing receptors are e.g. characterized by a fast deactivation and hence mediate short depolarization [70]. We observed that while MS increased and SS decreased NR2a mRNA expression, the combination of both stressors resulted in expression levels that were comparable to the ones observed in CON. Even though the enhanced NR2a expression seen in MS could be in line with the reduced mechanical sensitivity of these animals. Nevertheless, other sub-units such as NR2B, shown to play a crucial role in CFA-induced hyperalgesia should be included in the measurements [14,71].

Pharmacological activation of Group I metabotropic glutamate receptors such as mGluR 1 and 5 can elicit spontaneous nocifensive behaviors [72], potentiate the responses of AMPA and NMDA receptors [73] and play an important role for central sensitization during peripheral inflammation [74], their roles are however distinct. Indeed, mGluR1 and 5 agonists but not selective mGluR5 agonists have been shown to enhance spinothalamic tract (STT) cell responses to innocuous mechanical stimuli in monkeys undergoing capsaicin-induced inflammation [16]. On the other hand, selective agonists of mGluR5 have been shown to produce mechanical hyperalgesia while antagonists elicit a dose dependent reversal of CFA-induced inflammatory hyperalgesia [75]. Furthermore selective mGLuR5 antagonists induce a reversal of mechanical hyperalgesia in a CFA model of inflammation [76]. We did not observe any significant differences in mGluR5 gene (GRM5) expression in any of the stress groups during ongoing inflammation. Also, mGluR1 expression was only different between MS and the groups that underwent SS (SS and MS+SS). However, behavioral results collected after 21 days of inflammation indicated that MS animals only displayed significant differences to CON (mechanical thresholds) and MS+SS (thermal sensitivity). Consequently, mGluRs levels are difficult to relate to the pain-mediated behavior observed in the present study.

### Impact of stress and inflammation on astrocyte and microglial activation

Chronic stress has been shown to have an impact on microglia. Indeed, rats undergoing a similar protocol of MS (3h/days during the SHRP) as used in the present study displayed enhanced microglial activity [77]. At adult age, chronic restraint stress led to an increased number of Iba1-positive cells in various stress-sensitive brain regions [78] while chronic unpredictable stress induced an initial phase of activation and proliferation followed by apoptosis of hippocampal microglia cells [79]. Microglial cell activation is believed to be involved in the onset of inflammation-induced hypersensitivity. Indeed, following induction of CFA-induced paw inflammation microglial cells display a rapid and intense activation at the L5 level of the spinal cord as shown by Raghavendra and colleagues [18]. However this result is not always consistent following intra-plantar CFA injection [53,80]. In the present study we did not observe any differences in Iba1 mRNA expression between any of the groups 21 days after CFA-injection. This result could be due to two reasons 1) the various stressors might not have affected the spinal cord and 2) microglial activation could already have subsided at the late measurement time point after the onset of inflammation.

Similarly to microglial cells, astrocytes are sensitive to both early life and adult chronic stress, they do however display a more enduring activation following CFA injection. Indeed, their activation is sustained from day 4 to at least day 14 after CFA paw injection as seen by the GFAP and S100β astrocytic marker mRNA levels [18]. Furthermore, ELS induces a rapid (after 24h) downregulation of astrocytic markers GFAP and S100β in the anterior cingulate cortex (ACC), a crucial region for pain processing [81]. Also, chronic unpredictable stress or repeated administration of CORT lead to GFAP downregulation in the prefrontal cortex and hippocampus [82,83]. Here we showed that SS and the combination of MS and SS but not MS *per se* reduced GFAP mRNA expression in the spinal cord 21 days after CFA-injection. The reduced GFAP mRNA levels seen in SS and MS+SS could be indicative of an enhanced astrocyte reorientation, a reorganization of the astrocytic cytoskeleton (since GFAP indicates the reactivity and is involved in the maintenance of the structure of astrocytes). In order to determine whether our results represent a difference in astrocytic morphology or a reduction of the astrocyte population it would be necessary to complete our protocol with other markers such as S100β or other techniques such as immunostaining. Furthermore, astrocytes directly participate in the homeostasis of the glutamatergic synapse [65,84] and are able to modulate spinal nociceptive processing via the release of cytokines [18]. Concerning the MS and MS+SS animals this could be of particular interest as they respectively displayed different mechanical and thermal sensitivities 21 days after CFA injection.

### Alteration of pro-inflammatory cytokines by stress and inflammation

Clinical and preclinical settings often associate chronic stress exposure with major depressive disorders and enhanced pro-inflammatory cytokine levels, most consistently IL-1β and IL-6 [85,86]. Preclinical studies show that pro-inflammatory cytokines are mediating the long-term effects of chronic stress exposure [79,87–89]. We did not observe any significant differences between the 4 groups in any of the three cytokines measured after 21 days of inflammation. These results are surprising since TNF-α, IL-β and IL-6 expression in the spleen or plasma have been shown to be changed by social [90–93] and by post-natal stress [8,94]. We believe that the lack of differences in pro-inflammatory cytokine levels between groups could have two causes. Despite evidence supporting the ability of peripheral cytokines to signal through the blood brain barrier [95] it seems that spinal pro-inflammatory cytokine levels do not directly correlate with enhanced circulating cytokines [56]. Furthermore, microglia-induced immune activation and cytokine production are believed to be critical for the onset of arthritis [96,97], therefore differences to be expected during the initial phase of inflammation could have waned 21 days after CFA-injection.

### Influence of stress and inflammation on spinal NGF and GDNF levels

Neurotrophic factors are crucial for C-fiber nociceptor development and survival upon insults [98,99]. In this context, two C-fiber sub-classes are classified according to the neurotrophic factor affecting them. The tyrosine kinase A receptor (Trk A) expressing neurons depend on NGF while the Trk rearranged during transfection (Ret) neurons depend on GDNF. Spinal NGF has been shown to mediate neuronal plasticity involved in visceral hypersensitivity [100]. Also, socially stressed rats undergoing CCI displayed higher NGF levels and enhanced pain sensitivity than rats only undergoing CCI [51]. In our hands, MS and SS only had a tendency to increase NGF mRNA levels after 21 days of inflammation. This effect was not found in animals experiencing both stressors. However, 21 days after CFA-injection, MS+SS rats displayed longer nocifensive behavior latencies on the hot plate than MS and CON group. Hence, it is difficult to draw any explicit conclusions about a possible cause-effect relationship between NGF gene expression and stress-related behaviors.

GDNF delivered intrathecally has been shown to be a potent analgesic agent [101,102]. However, endogenous GDNF is believed to play a role in sensitization and the decrease of mechanical thresholds [103,104]. We did not observe any significant differences between any of the groups suggesting that GDNF was not involved in the CFA-induced central sensitization.

## Conclusion

This study aimed to test the commonly expressed assumption that the combination of early life stress and exposure to social stress at adulthood predisposes rats to affective disorders and comorbid pain states. However, rats exposed to the two stressors did not display clear alterations of mood and pain related behaviors. In addition, the measurement of biochemical markers of stress and/or pain processing did not reveal any congruent differences between the experimental groups. In spite of potential limitations related to sample size and measurement time points we believe that these negative results may allude to a more complicated interdependence of stress, affect and pain processing than initially assumed. Future studies should be aimed at a systematic evaluation of potentially relevant parameters.
